# Targeted therapy of rheumatoid arthritis via macrophage repolarization

**DOI:** 10.1080/10717544.2021.2000679

**Published:** 2021-11-12

**Authors:** Xu Zhou, Dandan Huang, Runkong Wang, Mingquan Wu, Liyang Zhu, Wei Peng, He Tu, Xuangeng Deng, He Zhu, Zhong Zhang, Xinming Wang, Xi Cao

**Affiliations:** a Sichuan Provincial Orthopedic Hospital, Chengdu, China; b Key Laboratory of Drug Targeting and Delivery Systems, Ministry of Education, West China School of Pharmacy, Sichuan University, Chengdu, China; c Department of Pharmacy, The First Affiliated Hospital of Anhui Medical University, Hefei, China

**Keywords:** Rheumatoid arthritis, macrophage repolarization, folate receptor, triptolide, liposomes

## Abstract

The polarization of macrophages plays a critical role in the physiological and pathological progression of rheumatoid arthritis (RA). Activated M1 macrophages overexpress folate receptors in arthritic joints. Hence, we developed folic acid (FA)-modified liposomes (FA-Lips) to encapsulate triptolide (TP) (FA-Lips/TP) for the targeted therapy of RA. FA-Lips exhibited significantly higher internalization efficiency in lipopolysaccharide (LPS)-stimulated RAW 264.7 cells than liposomes (Lips) in the absence of folate. Next, an adjuvant-induced arthritis (AIA) rat model was established to explore the biodistribution profiles of FA-Lips which showed markedly selective accumulation in inflammatory paws. Moreover, FA-Lips/TP exhibited greatly improved therapeutic efficacy and low toxicity in AIA rats by targeting M1 macrophages and repolarizing macrophages from M1 to M2 subtypes. Overall, a safe FA-modified liposomal delivery system encapsulating TP was shown to achieve inflammation-targeted therapy against RA via macrophage repolarization.

## 1. Introduction

Rheumatoid arthritis (RA) is a chronic autoimmune disease that is mostly characterized by chronic inflammation of the joint. The clinical symptoms include cartilage and bone damage and joint function deterioration, which eventually lead to disability. RA affects approximately 1% of the population worldwide and is more prevalent in women than in men; additionally, the affected population tends to be younger (McInnes & Schett, 2017; Tardito et al., 2019). Based on the fact that more than 90% of patients with RA could exhibit different degrees of disability within two decades of onset, causing a huge threat to the quality of life and security of RA patients, treating RA early is crucial (Emery et al., 2002). To deal with RA, current interventions mainly include medication, physiotherapy, and adjustments to lifestyle. For drug therapy, disease-modifying antirheumatic drugs (DMARDs), glucocorticoids (GCs), and nonsteroidal anti-inflammatory drugs (NSAIDs) are most commonly used to effectively improve this condition, but the long-term administration and the high-doses of these drugs may cause serious side effects. Therefore, complementary and alternative treatment options are greatly needed to improve the therapeutic effects and decrease side effects (Yuan et al., 2012; Wang et al., 2016; Ebel & O’Dell, 2021; Kapoor et al., 2021).

With in-depth research on the pathology of the inflammatory lesions of RA, it has been recognized that the extravasation through leaky vasculature and subsequent inflammatory cell-mediated sequestration (ELVIS) effect could allow nanoparticles of proper sizes to selectively accumulate in inflammatory sites (Crielaard et al., 2012; Wang et al., 2016; Zhou et al., 2019; Maity et al., 2021). For instance, Jia et al. successfully fabricated a novel liposome loaded with dexamethasone to achieve targeted therapy toward RA based on the ELVIS effect (Jia et al., 2018). In addition, since inflammatory cells pathologically overexpress specific receptors on the cell membrane, it is possible to correspondingly modify nanoparticles for targeted RA treatment (Yang et al., 2021).

Studies have shown that the number of macrophages in inflamed synovial tissue increases dramatically throughout the course of RA, and these cells play key roles in physiological and pathological responses (Yang et al., 2021). Macrophages in inflamed joints are classically activated and polarized into the M1 type and secrete a large number of proinflammatory cytokines, such as TNF-α, IL-1, and IL-6, to sustain inflammation (Jain et al., 2015; Wang et al., 2017; Feng & Guo, 2020; Yu et al., 2020). M2-type macrophages, another polarization phenotype of macrophages, on the other hand, can alleviate inflammation via the production of anti-inflammatory cytokines, including IL-10 and TGF-β (Tardito et al., 2019). In addition, macrophages overexpress pattern recognition receptors (PRRs) on their cell membrane, including folate receptors (FRs) (Hu et al., 2015; Yang et al., 2016; Wang et al., 2021). Importantly, M1-type macrophages overexpress FR only after activation, while quiescent macrophages and other inflammatory cells hardly express this receptor (Varghese et al., 2007; Nogueira et al., 2015; Mohammadi et al., 2016; Kottarath et al., 2020). Conjointly, targeting M1 macrophages and shifting M1 macrophages in arthritic joints to the M2 phenotype could be a promising strategy for the treatment of RA.

Triptolide (TP), a diterpene triepoxide isolated from *Tripterygium wilfordii* Hook F, has been widely used to treat inflammatory and autoimmune diseases, such as systemic lupus erythematosus and RA, but its severe systemic toxicity, especially hepatotoxicity, nephrotoxicity, and cardiotoxicity, along with poor solubility, results in a narrow therapeutic window and poor bioavailability that limit its clinical application (Shen et al., 2019; Song et al., 2020; Liaw et al., 2021; Liu et al., 2021). TP has been demonstrated to exhibit anti-inflammatory and immunosuppressive activities, and TP derivatives have been demonstrated to inhibit the polarization of macrophages into the proinflammatory M1 phenotype (Shen et al., 2019; Fu et al., 2020; Liaw et al., 2021; Liu et al., 2021). Folic acid (FA)-modified liposomes (FA-Lips) have been demonstrated to have enhanced targeting efficiency by binding to FR with high affinity (Nogueira et al., 2015; Xue et al., 2020). Moreover, liposomes (Lips) present excellent biodegradability and biocompatibility, thus making them suitable for the delivery of drugs *in vivo*. Therefore, FA-Lips are ideal carriers to entrap TP for synergistic and targeted RA treatment.

In this study, we report a novel approach to encapsulate TP in FA-modified liposomes (FA-Lips/TP) for targeted therapy of RA by inducing M1-to-M2 macrophage repolarization. To verify the condition of macrophages and FR expression in RA patients, immunofluorescence staining of synovial tissue samples from RA patients was performed. FA-Lips were characterized, and the cellular uptake efficiency of FA-Lips by LPS-stimulated RAW 264.7 cells was also evaluated. In addition, using adjuvant-induced arthritis (AIA) rats, biodistribution, and pharmacodynamic studies were investigated to evaluate the targeted therapeutic efficacy and safety of FA-Lips/TP.

## 2. Materials and methods

### 2.1. Materials

Triptolide was purchased from Chengdu Biopurify Phytochemicals Ltd. (Chengdu, China). 1,2-Distearoyl-sn-glycero-3-phosphoethanolamine-N-[methoxy (polyethylene glycol)] (DSPE-MPEG_2000_) and DSPE-PEG_2000_-folic acid (DSPE-PEG_2000_-FA) were provided by Ponsure Biological Ltd. (Shanghai, China). 1,1-Dioctadecyl-3,3,3′,3′-tetramethyl indodicarbocyanine (DiD) and lipopolysaccharides (LPS) were purchased from Sigma-Aldrich (St. Louis, MO). Lipoid E80 (purified ovolecithin) was obtained from Lipoid Co., Ltd. (Ludwigshafen, Germany). Cholesterol was purchased from Shanghai Aladdin Biochemical Technology Co., Ltd. (Shanghai, China). All other chemicals and reagents were of analytical grade.

### 2.2. Cell culture and animals

The murine macrophage cell line RAW 264.7 was obtained from the American Type Culture Collection (Gaithersburg, MD). The cells were cultured in Dulbecco's modified Eagle’s medium (DMEM) containing 10% fetal bovine serum (Gibco, Carlsbad, CA) and 1% penicillin/streptomycin (Solarbio, Beijing, China). The cells were cultured at 37 °C in a humidified atmosphere with 5% CO_2_.

Male Sprague-Dawley (SD) rats weighing 180–220 g were obtained from Chengdu Dossy Experimental Animals Co., Ltd. (Chengdu, China). All animal studies were approved by the Ethics Committee of Sichuan Provincial Orthopedic Hospital.

### 2.3. Immunofluorescence staining of macrophage and folate receptors in RA patient synovial tissue samples

Synovial tissue samples from RA patients which were embedded in paraffin were collected from the Pathology Department of the First Affiliated Hospital of Anhui Medical University. Ethics approval was obtained from the Ethics Committee of the First Affiliated Hospital of Anhui Medical University (no. 5101249) and informed consent was obtained from the patients. The samples were sectioned for F4/80 (macrophage marker) and FR immunofluorescence staining, and the cell nuclei were stained with DAPI. The samples were observed with LSCM.

### 2.4. Preparation and characterization of TP-loaded liposomes (Lips/TP) and TP-loaded folic acid-modified liposomes

Lips/TP were fabricated by a thin-film hydration method (Jia et al., 2018). Specifically, 50 mg of E80, 10 mg of cholesterol, 5 mg of DSPE-MPEG_2000_, and 1.5 mg of TP were dissolved in 20 mL of chloroform and evaporated under vacuum at 30 °C for 6 min by a rotary evaporator. The resultant thin film was hydrated with 5 mL of ultrapure water at 37 °C and then sonicated for 3 min in an ice bath to prepare the Lips/TP. FA-Lips/TP were fabricated similarly by using DSPE-MPEG_2000_-FA instead of DSPE-MPEG_2000_.

To characterize Lips/TP and FA-Lips/TP, the particle sizes and zeta potentials were determined by dynamic light scanning (DLS) analysis (Malvern, Nano ZS90, Malvern, UK), and morphology was determined by transmission electron microscopy (TEM) (Hitachi, H-600, Tokyo, Japan). To investigate stability, Lips/TP and FA-Lips/TP were placed at room temperature of 25 °C for 48 h. Particle size distribution was recorded at the given time of 0, 2, 4, 8, 24, and 48 h.

### 2.5. Encapsulation efficiency, loading capacity, and *in vitro* release of Lips/TP and FA-Lips/TP

To measure the encapsulation efficiency (EE) and loading capacity of TP, certain volumes of Lips/TP and FA-Lips/TP were centrifuged at 6500 rpm for 30 min separately using an ultrafiltration tube (Mw = 10 kDa, Millipore, Billerica, MA) (Li et al., 2015; Wang et al., 2017; Jia et al., 2018). The level of TP in the filtrate was determined by high-performance liquid chromatography (HPLC, Waters, Milford, MA). A Waters Symmetry^®^ C18 column (4.6 mm × 150 mm, 5 μm) was adopted, the mobile phase consisted of acetonitrile and water (26:74) at a flow rate of 1.0 mL/min, the column temperature was 30 °C, and the detection wavelength was 218 nm. Equal volumes of Lips/TP and FA-Lips/TP were dissolved in methanol, and the total levels of TP in the preparations were determined. The EE and drug loading (DL) capacity efficiency of TP were calculated by the following equations:

$$\mathrm{EE}\;(\%)=\frac{\text{weight of encapsulated drugs}}{\text{total weight of drugs}}\times 100\%$$

$$\mathrm{DL}\;(\%)=\frac{\text{weight of encapsulated drugs}}{\text{total weight of liposomes}}\times 100\%$$

The *in vitro* release of TP from Lips and FA-Lips was investigated using the dialysis method. In brief, 2 mL of Lips was added to dialysis bags (MW = 3500 Da, Solarbio, Beijing, China) and subjected to dialysis with 45 mL of PBS containing 0.1% Tween 80. Free TP solution was used as the control. The solutions were stirred at 100 rpm at 37 °C with a thermostat water bath vibrator. At the predetermined time points, 1 mL of release medium was collected and replaced with an equal volume of fresh release medium. The concentration of TP at each time was determined by HPLC, and the cumulative drug release percentage was calculated accordingly (Cao et al., 2016).

### 2.6. Cellular uptake assay

To evaluate the cellular uptake efficiency of the Lips, the hydrophobic infrared fluorescent dye DiD was encapsulated in Lips (Lips/DiD and FA-Lips/DiD). RAW 264.7 cells were seeded in 12-well plates (1 × 10^5^ cells per well) for 24 h. Then, the cells were pretreated or not pretreated with LPS (2 μg/mL) for 4 h and washed with PBS three times. Next, the cells were incubated with Lips/DiD and FA-Lips/DiD (an equivalent dose of 0.5 μg/mL DiD) for an additional 0.5 h and 2 h. Then, the medium was removed, and the cells were washed with PBS three times and harvested. The fluorescence intensity of DiD in the cells was analyzed by flow cytometry (BD FACSCelesta, San Jose, CA).

For qualitative analysis of uptake by LPS-stimulated RAW 264.7 cells, the cells were seeded in glass bottom dishes (2 × 10^4^ cells per dish) and stimulated with LPS as described previously. After being incubated with Lips/DiD and FA-Lips/DiD for 0.5 and 2 h, the cells were fixed in 4% paraformaldehyde for 20 min, followed by DAPI staining for 5 min. The samples were then examined by laser scanning confocal microscopy (LSCM, LSM 800, Zeiss, Oberkochen, Germany).

Next, the cellular uptake of Lips/TP and FA-Lips/TP by LPS-stimulated RAW 264.7 cells was also evaluated as mentioned before. After harvesting the cells, they were lysed by three repeated freeze–thaw cycles. Then, the suspension was collected for the protein measurement by the enhanced bicinchoninic acid protein assay kit and TP quantitation by liquid chromatography–tandem mass spectrometry as described previously (Zhang et al., 2013; Zhou et al., 2014; Fu et al., 2016). The cellular uptake was expressed as the amount (ng) of TP associated with a unit weight (mg) of cellular protein.

### 2.7. *In vitro* anti-inflammatory efficiency

RAW 264.7 cells were seeded in 12-well plates (1 × 10^5^ cells per well) for 24 h. Then, the cells were pretreated with LPS (2 μg/mL) for 4 h, and incubated with different preparations for another 24 h. Cells were then collected and the mRNA levels of TNF-α and IL-1β were determined by reverse transcriptase-polymerase chain reaction (RT-PCR) (Wang et al., 2017).

### 2.8. Establishment of the adjuvant-induced arthritis rat model

The AIA rat model was established according to a previous report (Wang et al., 2016). SD rats were subcutaneously injected at the base of the tail with 0.1 mL of 10 mg/mL complete Freund’s adjuvant. Arthritis progression was monitored daily, and rats with obvious inflammation and swollen paws were selected as AIA rats at 2 weeks after adjuvant injection.

### 2.9. *In vivo* biodistribution of FA-Lips in AIA rats

The DiD solution, Lips/DiD, and FA-Lips/DiD (an equivalent dose of 70 μg/kg DiD) were administered to AIA rats via the tail vein. The rats were anesthetized at 2, 6, and 24 h after injection and visualized by an IVIS Lumina III In Vivo Imaging System (PerkinElmer, Waltham, MA). Then, the rats were sacrificed, and their organs and hind legs were collected for *ex vivo* imaging (Zhou et al., 2017).

### 2.10. Pharmacodynamics analysis

#### 2.10.1. Paw thickness and paw volume measurement in AIA rats

AIA rats were randomly divided into four groups (*n* = 6): the saline group, TP solution group, Lips/TP group, and FA-Lips/TP group. The rats were injected intravenously with different formulations (an equivalent dose of 50 μg/kg TP) every other day five times (Li et al., 2020; Liu et al., 2021). Untreated rats without adjuvant induction were used as controls. Paw thickness in the rear limbs of rats was measured on day 14, 16, 18, 20, 22, and 24 after arthritis induction by a Vernier caliper. To ensure the accuracy of the measurement, we measured the paw thickness from the arch of the rat paws every time. Paw volumes were measured by a paw volume meter.

#### 2.10.2. Pro-inflammatory cytokines expression

After the final treatment administration and paw thickness measurement, the rats were sacrificed, and blood and joint tissues were collected. The concentrations of pro-inflammatory cytokines in blood, including TNF-α and IL-1β, were quantitated using ELISA kits according to the manufacturer’s instructions. Additionally, the mRNA levels of TNF-α and IL-1β in joint tissues were determined by RT-PCR (Wang et al., 2017).

#### 2.10.3. Histological analysis of ankle joints

The rear limbs of rats in all groups were collected after sacrifice, fixed in 4% paraformaldehyde for 24 h, decalcified by immersion in a 15% neutral EDTA solution for 14 days, and then embedded in paraffin for pathological sectioning. The sections were stained with hematoxylin–eosin (H&E) to evaluate the therapeutic effect of the formulations.

#### 2.10.4. Immunofluorescence staining of M1 and M2 macrophages

The ankle joint sections were stained with anti-iNOS (M1 macrophage marker) and anti-CD206 (M2 macrophage marker) simultaneously. Then, nuclei were stained with DAPI. The samples were observed by LSCM.

### 2.11. Safety evaluation

The body weights of AIA rats were measured on day 14, 16, 18, 20, 22, and 24 after arthritis induction. Then, the rats were sacrificed, and vital organs, including the heart, liver, spleen, lung, and kidney, were collected. All organs were fixed with 4% paraformaldehyde and embedded in paraffin. Next, the samples were subjected to H&E staining to assess the pathological characteristics. Serum alanine transaminase (ALT), aspartate aminotransferase (AST), blood urea nitrogen (BUN), and creatine (Cre) levels were measured to evaluate the hepatotoxicity and nephrotoxicity of the preparations.

### 2.12. Statistical analysis

All quantitative data are presented as the mean and standard deviation (SD). Statistical analysis was performed by one-way ANOVA to compare multiple groups using GraphPad Prism software (La Jolla, CA). A *p* value of <0.05 was accepted as statistically significant.

## 3. Results

### 3.1. Co-localization of macrophages and folate receptors in synovial tissues from RA patients

**Figure 1.**
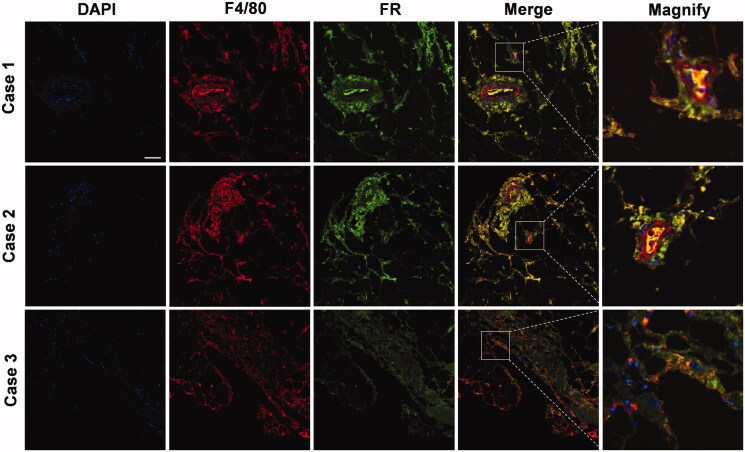
Immunofluorescence staining of macrophage (F4/80) and folate receptor (FR) expression from synovial tissue samples of RA patients. Scale bar represents 100 µm.

To confirm whether macrophages in synovial tissues from RA patients overexpress FRs, we managed to obtain three synovial tissue samples from RA patients and examined the level of FR expression in macrophages by immunofluorescence staining. As shown in Figure 1, considerable green fluorescence and red fluorescence were observed, and there was notable overlap. These results indicated that inflamed synovial tissues from RA patients were infiltrated with many macrophages overexpressing FRs, which was consistent with the observations of RAW 264.7 cells and AIA rats (Nogueira et al., 2016; Yang et al., 2021).

### 3.2. Fabrication and characterization of liposomes

**Figure 2.**
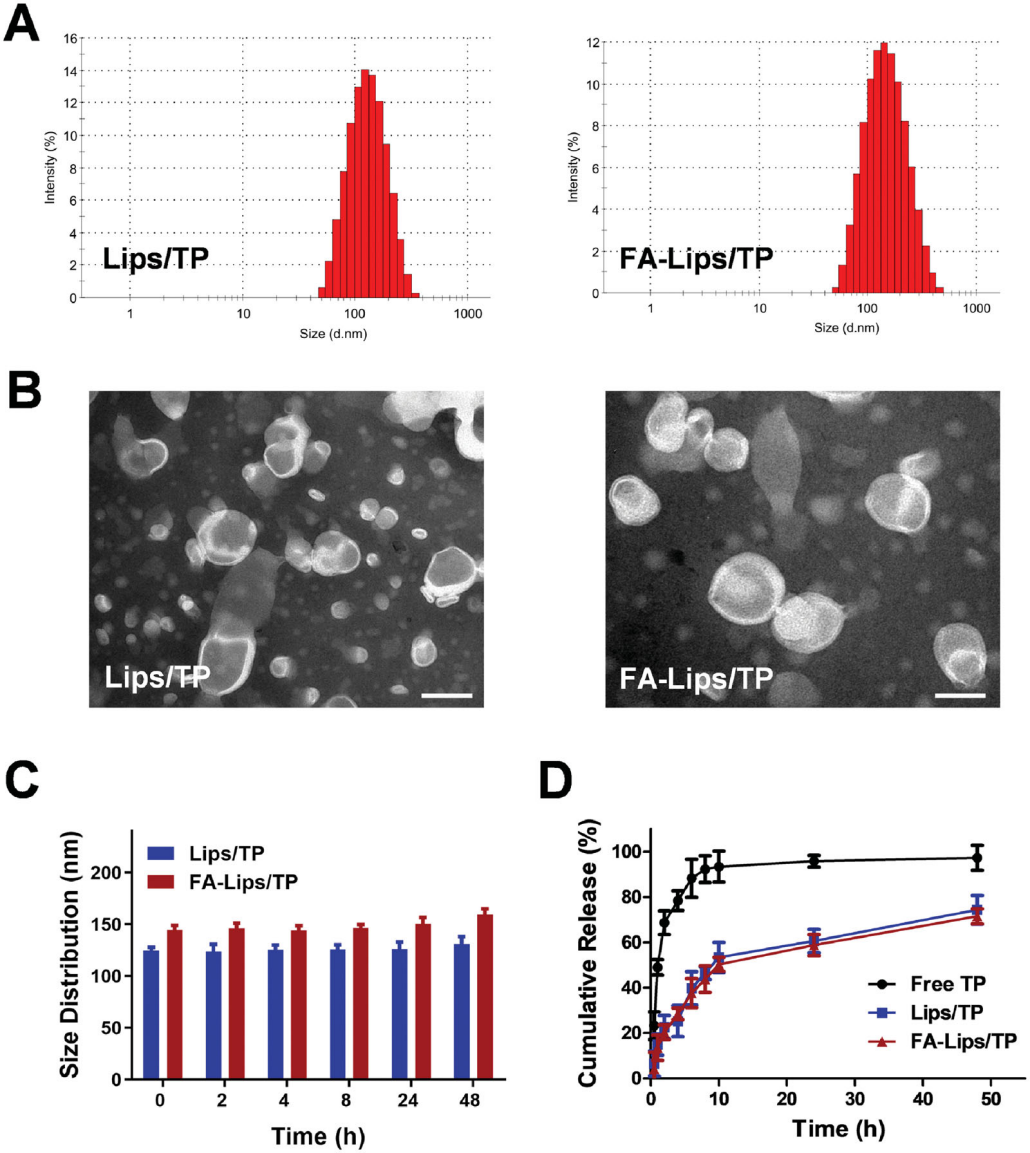
Characterization of liposomes. (A) Dynamic light scattering (DLS) size measurement of Lips/TP and FA-Lips/TP. (B) TEM images of Lips/TP and FA-Lips/TP. (C) Size distributions of Lips/TP and FA-Lips/TP at the given time by DLS. (D) *In vitro* release of Lips/TP and FA-Lips/TP. Scale bar represents 100 nm.

Lips/TP and FA-Lips/TP were successfully fabricated by the thin-film hydration method. As shown in Figure 2(A), both Lips/TP and FA-Lips/TP displayed homogeneous particle sizes with normal distributions. Specifically, Lips/TP showed an average size of 124.7 nm with a polydispersity index (PDI) of 0.236. FA-Lips/TP, on the other hand, showed a slight size increase of approximately 20–144.5 nm (PDI = 0.253) (Table 1). The TEM results illustrated that both types of Lips displayed uniform spherical shapes with lipid bilayer structures, which are typical liposome morphological characteristics (Figure 2(B)). In addition, the zeta potential of Lips/TP was −14.3 ± 2.1 mV, and FA-Lips/TP showed an average surface charge of −20.7 ± 3.6 mV, which was slightly decreased compared to that of Lips/TP. This result was likely due to the FA, which has a negative charge, modification on the liposome surface. The average liposome size was nearly unchanged after storage at 25 °C for 48 h, indicating that the formulations of Lips were stable for the tested time (Figure 2(C)).

**Table 1.** Characterization of Lips/TP and FA-Lips/TP.

Liposomes	Size (nm)	PDI	Zeta potential (mV)
Lips/TP	124.7 ± 3.1	0.236 ± 0.050	–14.3 ± 2.1
FA-Lips/TP	144.5 ± 4.6	0.253 ± 0.016	–20.7 ± 3.6

Data represent mean ± SD (*n* = 3).

### 3.3. Encapsulation efficiency, loading capacity, and *in vitro* release of liposomes

After the Lips were characterized, the EE and DL efficiency were also examined. The EE (%) of Lips/TP was approximately 85.3%, and the DL (%) was 1.8%. TP was incorporated into FA-Lips with an EE of 90.3% and DL efficiency of 2.2% (Table 2).

**Table 2.** Encapsulation efficiency and loading capacity of liposomes for triptolide encapsulation.

Liposomes	EE (%)	DL (%)
Lips/TP	80.3 ± 2.4	1.8 ± 0.2
FA-Lips/TP	84.3 ± 3.6	2.1 ± 0.3

Data represent mean ± SD (*n* = 3).

As shown in Figure 2(D), free TP solution was rapidly released, with up to 92.3% release after 8 h. In contrast, encapsulated TP in Lips and FA-Lips was released slowly. Specifically, approximately 61% of TP was released from Lips, and 59% of TP was released from FA-Lips after 24 h, indicating relatively low leakage of TP at the condition of 37 °C and pH 7.4.

### 3.4. Cellular uptake and anti-inflammatory efficiency of liposomes

**Figure 3.**
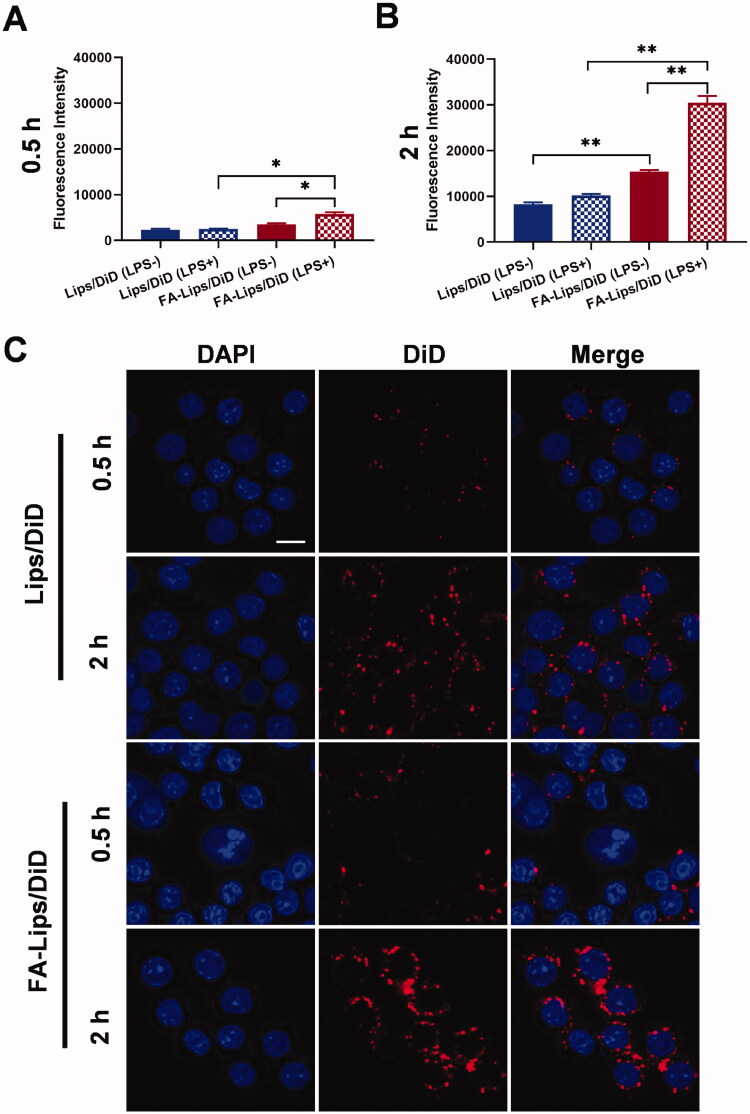
Cellular uptake behaviors of liposomes. (A) Cellular uptake of Lips/DiD and FA-Lips/DiD in normal macrophage RAW 264.7 cells and LPS-stimulated RAW 264.7 cells at 0.5 h. (B) Cellular uptake of Lips/DiD and FA-Lips/DiD in normal macrophage RAW 264.7 cells and LPS-stimulated RAW 264.7 cells at 2 h. (C) Laser scanning confocal microscopy (LSCM) images of cellular uptake in LPS-stimulated RAW 264.7 cells. Scale bar represents 10 μm. *p < 0.05; **p < 0.01.

The level of FR expression on LPS-stimulated cells was examined by immunofluorescence staining. The results showed obvious upregulation of FR in RAW 264.7 cells after LPS stimulation (Figure S1). To evaluate cellular uptake efficiency, the internalization of Lips/DiD and FA-Lips/DiD by RAW 264.7 cells was measured by flow cytometry and LSCM. DiD was used as a fluorescent probe. Based on the flow cytometry results, the uptake of both Lips/DiD and FA-Lips/DiD by normal RAW 264.7 cells (LPS–) and LPS-stimulated RAW 264.7 cells (LPS+) increased in a time-dependent manner (Figure 3). In addition, the cellular uptake efficiency of FA-Lips/DiD was higher than that of Lips/DiD at the indicated time points, especially when the macrophages were activated (*p* < 0.05). Specifically, the fluorescence intensity of FA-Lips/DiD was 2.3 times higher than that of Lips/DiD at 0.5 h (*p* < 0.05) and 3.0 times higher at 2 h (*p* < 0.01) when RAW 264.7 cells were stimulated with LPS. The LSCM results also showed that the fluorescence intensity of FA-Lips/DiD in activated macrophages was apparently higher than Lips/DiD (Figure 3(C)), which was consistent with the quantitative results. In contrast, the uptake efficiency of Lips in mouse fibroblast 3T3-L1 cells, which do not express FR (Nigro et al., 2020), was extremely low compared to that in RAW 264.7 cells, and the fluorescence intensity of Lips/DiD and FA-Lips/DiD was hardly different (Figure S2). Moreover, the uptake of Lips/TP and FA-Lips/TP was significantly higher than TP at 0.5 h and 2 h (*p* < 0.01), and the uptake of FA-Lips/TP was higher than Lips/TP at 2 h (*p* < 0.05) (Figure S3). These results indicated the specific high uptake of FA-Lips by activated macrophages due to the specific interaction between FA and FR.

In addition, the cytotoxicity of the formulations in RAW 264.7 cells was also evaluated (Zhou et al., 2019). The results showed that whether Lips/TP or FA-Lips/TP could reduce the toxicity of TP (Figure S4).

Next, the pro-inflammatory cytokines expression of TNF-α and IL-1β in LPS-stimulated RAW 264.7 cells was investigated after incubating with the preparations. Although free TP could slightly reduce TNF-α and IL-1β levels, Lips/TP and FA-Lips/TP more significantly reduced TNF-α and IL-1β levels (*p* < 0.05) (Figure S5), indicating remarkable anti-inflammatory effect *in vitro*.

### 3.5. Targeted biodistribution of FA-Lips in AIA rats

**Figure 4.**
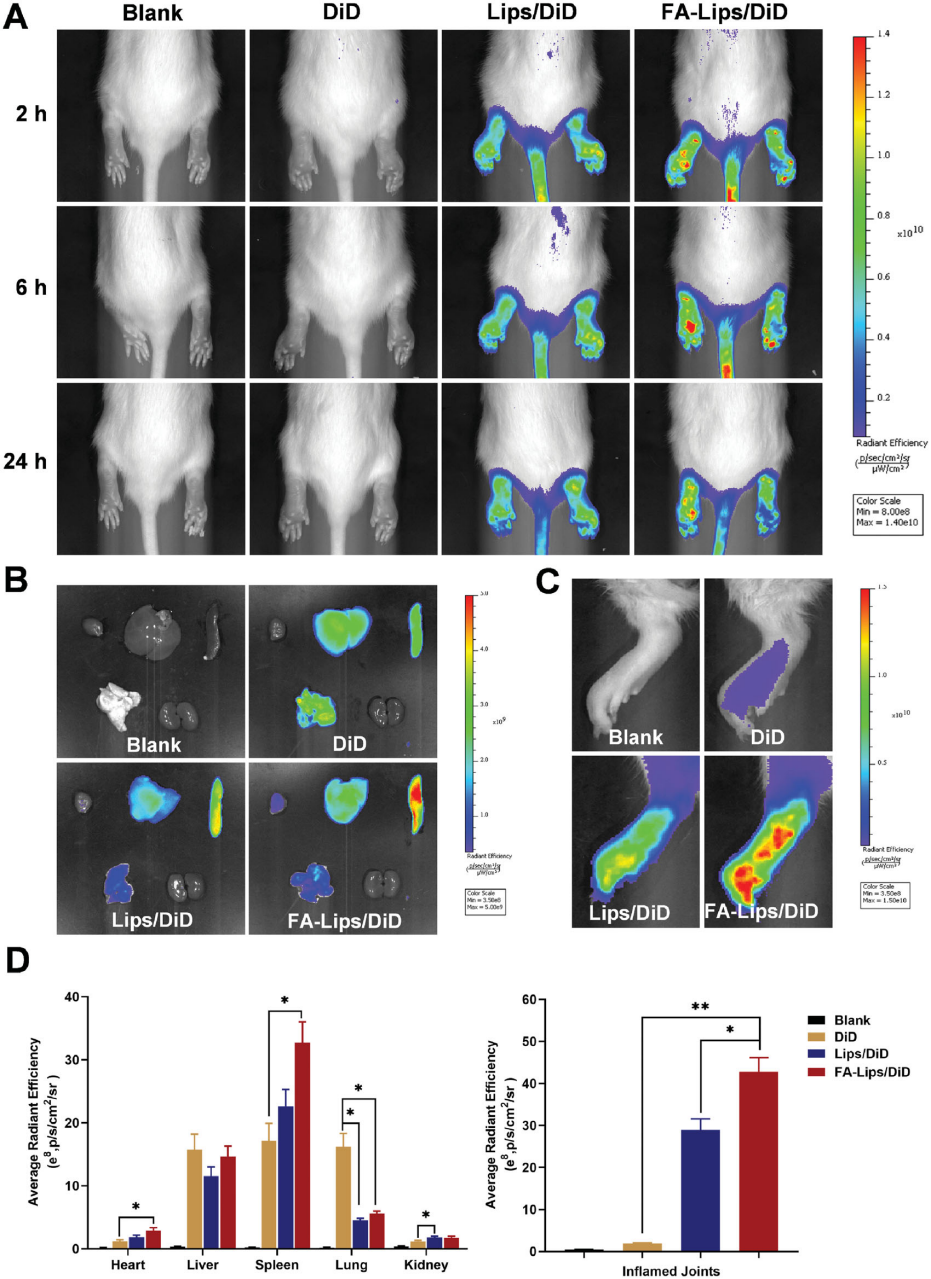
Biodistribution of Did loaded liposomes in AIA rats. (A) *In vivo* fluorescent imaging of DiD, Lips/DiD, and FA-Lips/DiD in AIA rats at 2 h, 6 h, and 24 h. (B) *Ex vivo* fluorescent imaging of major organs from AIA rats at 24 h. (C) *Ex vivo* fluorescence imaging in the inflamed joints from AIA rats at 24 h. (D) Semiquantitative analysis of fluorescent intensity within the organs and joints. *p < 0.05; **p < 0.01.

The *in vivo* biodistribution of the Lips is shown in Figure 4(A). No obvious fluorescence was observed in the DiD group. Both Lips/DiD and FA-Lips/DiD exhibited strong fluorescence in the rear limbs of AIA rats compared to that of the DiD solution. Importantly, FA-Lips/DiD more selectively accumulated in the inflamed joints than Lips/DiD at 2 h, 6 h, and 24 h. The *ex vivo* biodistribution in major organs is shown in Figure 4(B,D), and the liver and lung had the greatest fluorescence after 24 h in the DiD solution group. In contrast, more Lips seemed to accumulate in the spleen compared to the DiD solution. There was massive fluorescence accumulation in the limbs in the FA-Lips/DiD group after 24 h compared with the DiD solution group and Lips/DiD group (Figure 4(C,D)), indicating a prolonged circulation time and increased inflamed joint targeting efficiency.

### 3.6. Therapeutic efficacy of FA-Lips/TP in AIA rats

**Figure 5.**
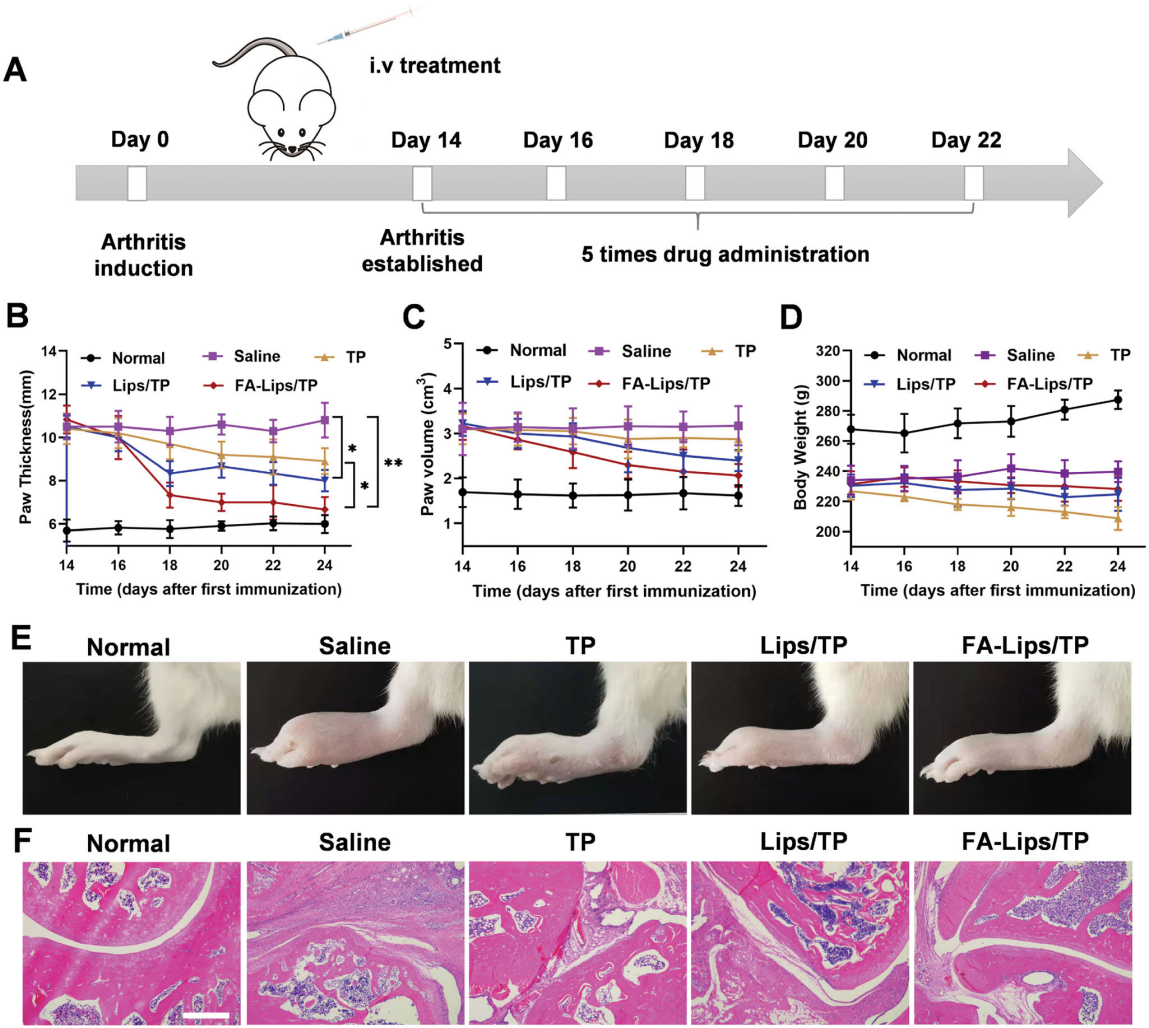
Therapeutic efficacy of FA-Lips/TP in AIA rats. (A) The schematic illustration of FA-Lips/TP treatment. (B) Paw thickness, (C) paw volume, and (D) body weight of AIA rats recorded after treatment. (E) Photographs of hindlimbs from different treatment groups. (F) Histopathology of ankle joints by H&E staining after treatment. Scale bar represents 200 μm. *p < 0.05; **p < 0.01.

The therapeutic efficacy of FA-Lips/TP for RA treatment was evaluated in an established AIA rat model. Paw swelling degree is one of most important indicators when evaluating the curative effect. After five injections of the preparations, the paw thicknesses in the Lips/TP and FA-Lips/TP groups were smaller than those in the saline treatment group (*p* < 0.05), while the TP solution treatment group only showed a slight decrease in paw thickness compared to that in the saline treatment group (Figure 5(B)). In addition, FA-Lips/TP decreased paw thickness and paw volume and more robustly improved the inflamed limbs than TP or Lips/TP (Figure 5(B,C,E)).

H&E staining of ankle joints in the saline group showed severe articular cartilage erosion and synovial inflammation compared to those of normal rats (Figure 5(F)). The rats treated with TP solution exhibited slightly reduced symptoms, while rats in the FA-Lips/TP group showed the largest reductions in cartilage damage and synovial inflammation compared to those in the Lips/TP and TP solution groups.

**Figure 6.**
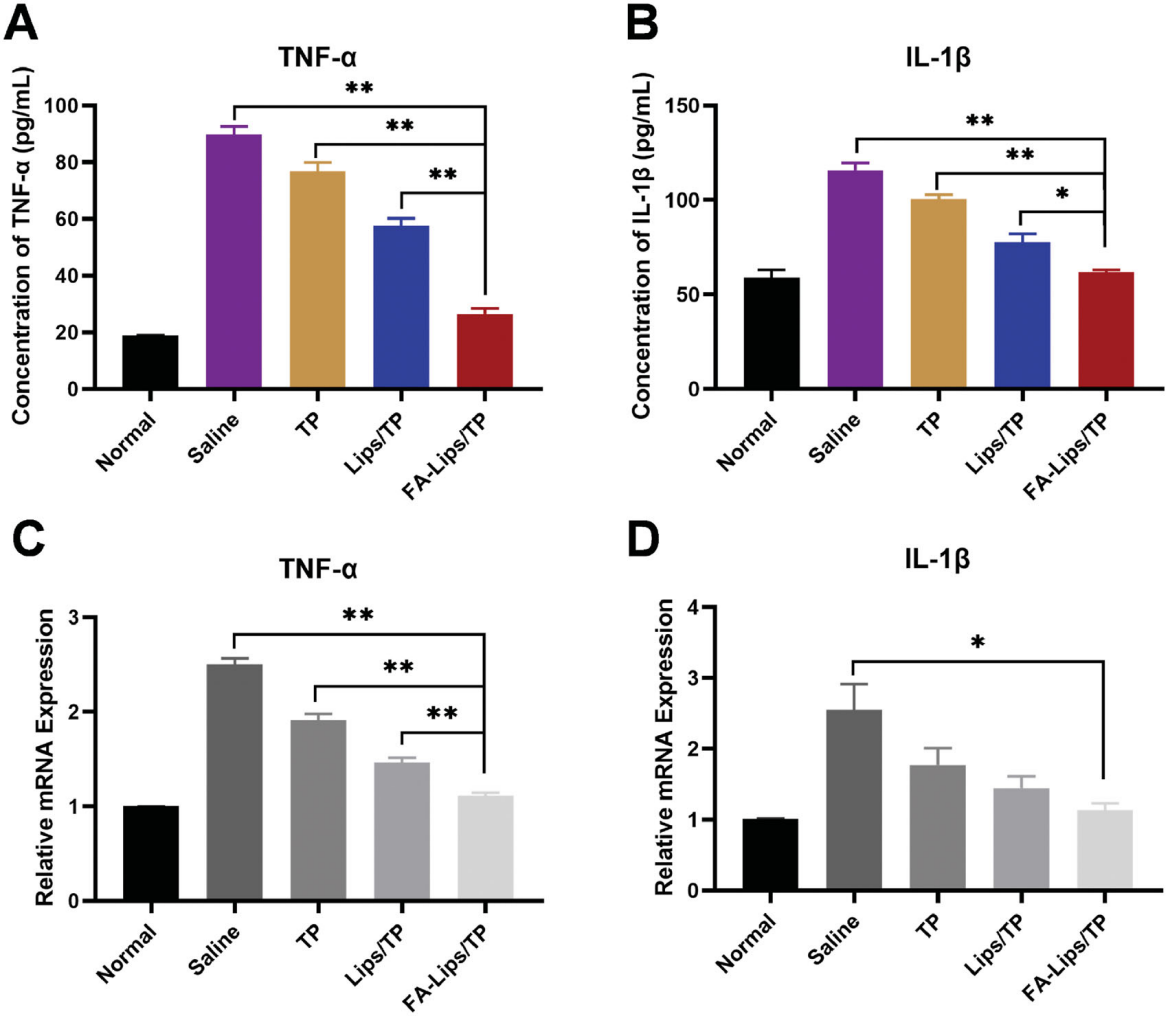
Pro-inflammatory cytokines expression in AIA rats after treatment. (A, B) TNF-α and IL-1β levels in blood measured by ELISA. (C, D) TNF-α and IL-1β levels in joint tissues measured by PCR. *p < 0.05; **p < 0.01.

In addition, the levels of inflammatory cytokines in blood and joint tissues were also measured to evaluate therapeutic efficacy. As shown in Figure 6(A,B), TNF-α and IL-1β levels in blood were significantly decreased in the FA-Lips/TP-treated group (*p* < 0.05) compared with the Lips/TP-, TP-, and saline-treated groups. Similar results were observed in the levels of these cytokines in joint tissues (Figure 6(C,D)), which was consistent with the paw swelling evaluation results.

**Figure 7.**
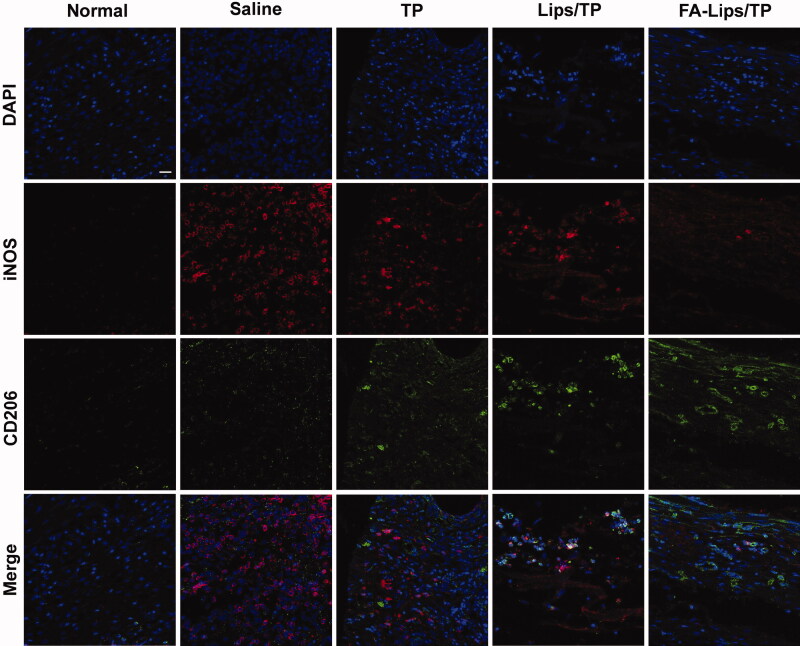
Immunofluorescence staining of M1 macrophage (iNOS, red) and M2 macrophage (CD206, green) markers in RA joints after treatment. Scale bar represents 20 μm.

Next, the repolarization of macrophages in ankle joints was assessed by immunofluorescence staining. As shown in Figure 7, strong red fluorescence (iNOS, M1 marker) and weak green fluorescence (CD206, M2 marker) in AIA rats were observed, which was consistent with the pathological features of RA. Both the Lips/TP and FA-Lips/TP groups showed higher levels of M2 macrophages and lower levels of M1 macrophages than the TP group. Furthermore, the most obvious change in M1 to M2 repolarization occurred in the FA-Lips/TP group, indicating the strongest anti-inflammatory effect among the groups.

### 3.7. Safety evaluation of FA-Lips/TP

**Figure 8.**
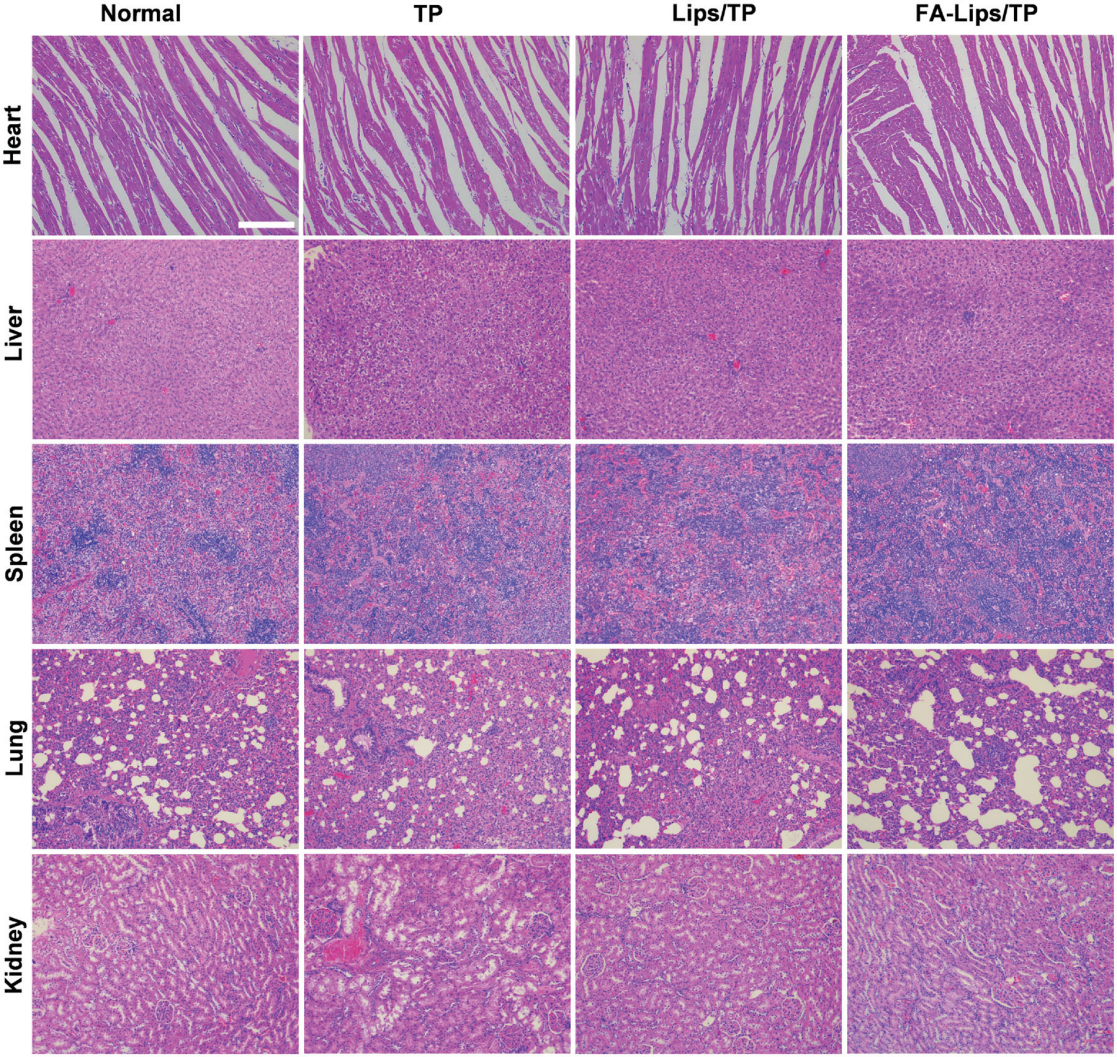
Histological analysis of H&E staining tissues in AIA rats after treatment. Scale bar represents 100 μm.

Changes in the body weights of AIA rats during the treatment were measured to evaluate safety. Compared to those in the saline group, rats treated with the TP solution showed obvious decreases in body weight, while the body weights in the Lips/TP and FA-Lips/TP groups remained almost unchanged (Figure 5(D)). Next, the pathological characteristics of the major organs were evaluated. As shown in Figure 8, the TP solution induced severe hepatic vacuolization, cytoplasmic deficiency, and renal tubular structural disorder. In addition, ALT, AST, and Cre levels in the serum of the TP group were significantly higher than those in the normal group (*p* < 0.05), and BUN levels were also higher than those in the normal group (Figures S6 and S7), indicating severe hepatotoxicity and nephrotoxicity. In contrast, neither the Lips/TP nor FA-Lips/TP groups showed obvious signs of hepatotoxicity or nephrotoxicity, indicating a reduction in the systemic toxicity of TP.

## 4. Discussion

Although there has been an increase in the mortality rate in the RA population due to relevant cardiovascular diseases, obesity and dyslipidemia, effective clinical treatments for RA are still limited (Avina-Zubieta et al., 2012; Naerr et al., 2016). The drugs presently used, including DMARDs and GCs, induce serious side effects. Biological agents such as adalimumab, a TNF inhibitor, and tocilizumab, an IL-6 inhibitor, have the relative disadvantages of high financial costs (Law & Taylor, 2019). Hence, alternative RA treatments with few side effects and relatively proper cost are essential and urgently needed.

The presence of a large number of macrophages is an obvious feature of inflammatory disease, and there is a direct correlation between the level of macrophage activity and the severity of RA during RA development (Mohammadi et al., 2016). Activated M1 macrophages produce multiple proinflammatory cytokines to sustain and exacerbate joint inflammation, while activated M2 macrophages secrete anti-inflammatory cytokines to resist this condition (Jain et al., 2015; Feng & Guo, 2020; Zhu et al., 2021). Hence, repolarizing activated macrophages from the M1 to M2 phenotype could be an effective strategy for RA treatment.

The traditional Chinese medicine *Tripterygium wilfordii* Hook F has demonstrated satisfactory therapeutic efficacy for RA patients in long-term clinical practice (Zhang et al., 2013). Triptolide, one of the active components of this medicine, has been suggested to have promising anti-inflammatory and immunosuppressive properties but is limited by its severe systemic toxicity (Xi et al., 2017). To improve the curative effect and reduce systemic toxicity, modified Lips are ideal carriers of TP because of their specific structure and functions (Du et al., 2015). Given that activated M1 macrophages localized to the pathological region of RA overexpress FRs, we successfully designed and fabricated FA-Lips to encapsulate TP, which is hydrophobic, into the lipid layer with a high EE.

In this study, the particle sizes of FA-Lips/TP were slightly increased by ∼20 nm compared to that of unmodified Lips/TP, and the zeta potential was also slightly decreased, which was very likely because of the negative FA modification on the surface of the Lips. Both Lips/TP and FA-Lips/TP displayed good stability and relatively slow and sustained release *in vitro*, which are critical characteristics of nanoparticle systems that target, extravasate, and accumulate in RA joints (van den Hoven et al., 2011).

Next, we found that the uptake of FA-Lips/DiD or FA-Lips/TP by LPS-stimulated macrophages was highly increased compared to that of Lips/DiD or Lips/TP. Since most normal cells/tissues except kidneys and hematopoietic tissue express very little FR, activated M1 macrophages overexpressing FR in arthritic joints allowed for the selective targeting of the folate-linked nanoparticle system to inflamed sites (Xia et al., 2009). Thus, FA-Lips had a higher affinity for macrophages than normal Lips, which resulted in significantly higher uptake efficiency. Additionally, we confirmed that the overexpression of FR in activated RAW 264.7 cells was consistent with FR expression in synovial tissues from RA patients, which was the basis of the design of FA-Lips. Moreover, the biodistribution results demonstrated that FA-Lips exhibited relatively selective accumulation in the inflamed joints with long circulation times, which was consistent with the cellular uptake results, indicating that Lips with the FA modification exhibited enhanced efficacy *in vitro* and *in vivo* by targeting M1-type macrophages in inflamed joints.

Previous studies have shown that nanoparticle-encapsulated TP could enhance the therapeutic effect on RA (Li et al., 2020; Liu et al., 2021). In our study, except that FA-Lips/TP showed increased anti-inflammatory effect *in vitro*, they also markedly decreased the degree of paw swelling in AIA rats compared to normal Lips/TP or the TP solution. In addition, the levels of TNF-α and IL-1β in both blood and joint tissues showed significant decreases in response to FA-Lips/TP. Since activated M1 macrophages mainly secrete the pro-inflammatory cytokines TNF-α and IL-1β, which are important parameters to assess therapeutic effects, the decrease in these pro-inflammatory cytokines indicated a reduction in M1 macrophages or the induction of the repolarization of M2 macrophages. Furthermore, the evaluation of macrophage phenotype distribution in ankle joints also demonstrated that FA-Lips/TP could effectively repolarize pro-inflammatory M1 macrophages to the anti-inflammatory M2 phenotype, demonstrating the strongest anti-inflammatory effect for RA treatment.

The clinical application of TP is restricted due to its narrow therapeutic window and side effects, especially hepatotoxicity and nephrotoxicity (Yuan et al., 2019; Xie et al., 2020). Given the safety concerns about the clinical use of TP, we demonstrated that FA-Lips/TP showed no obvious signs of hepatotoxicity or nephrotoxicity, which was likely due to the targeting of FA-Lips, leading to reduced distribution in other organs and explaining their relatively low toxicity.

Notably, the discovery of FR expression on macrophages in synovial tissues from human RA patients has demonstrated pathologic consistency with AIA animal models, increasing the possibility of applying FA-Lips/TP as a targeted therapy for RA clinically (Paulos et al., 2004).

## 5. Conclusions

In summary, we successfully developed facile TP-loaded FA-Lips for targeted RA treatment. FA-Lips demonstrated highly efficient cellular uptake *in vitro* and excellent therapeutic efficacy for RA *in vivo*. Specifically, activated M1 macrophages were proven to be important cellular targets for RA treatment. The reduction in M1 macrophages and the repolarization of macrophages from M1 to M2 phenotypes were critical in alleviating inflammation in arthritic joints. TP-loaded FA-Lips also showed significantly reduced systemic toxicity. Taken together, these findings suggest that targeting activated macrophages in the joint microenvironment represents a promising strategy for the treatment of RA.

## Supplementary Material

Supplemental Material
