# Supplementary material for: Targeted therapy of rheumatoid arthritis via macrophage repolarization
Source: Drug Deliv. 2021 Nov 12;28(1):2447–59. doi: 10.1080/10717544.2021.2000679 (PMC8592611; doi:10.1080/10717544.2021.2000679)
Supplement: Supplemental Material [file IDRD_A_2000679_SM1419.docx]

**Supporting Information**

**Targeted Therapy of Rheumatoid Arthritis via Macrophage Repolarization**

Xu Zhou^1, *^, Dandan Huang^2^, Runkong Wang^1^, Mingquan Wu^1^, Liyang Zhu^1^, Wei Peng^1^, He Tu^1^, Xuangeng Deng^1^, He Zhu^1^, Zhong Zhang^1^, Xinming Wang ^3^, Xi Cao^3,^ ^*^

^1^ Sichuan Provincial Orthopedic Hospital, Chengdu, 610041, China

^2^ Key Laboratory of Drug Targeting and Delivery Systems, Ministry of Education, West China School of Pharmacy, Sichuan University, Chengdu, 610041, China

^3^ Department of Pharmacy, The First Affiliated Hospital of Anhui Medical University, Hefei, 230000, China

* Correspondence: Xu Zhou and Xi Cao

Adress: No.132, the west 1st section of Yihuan Road, Chengdu 610041, China

Tel: +86-28-87050716 (Xu Zhou), +86-0551-62922423 (Xi Cao)

Email: 838677724@qq.com (Xu Zhou), 522292581@qq.com (Xi Cao)


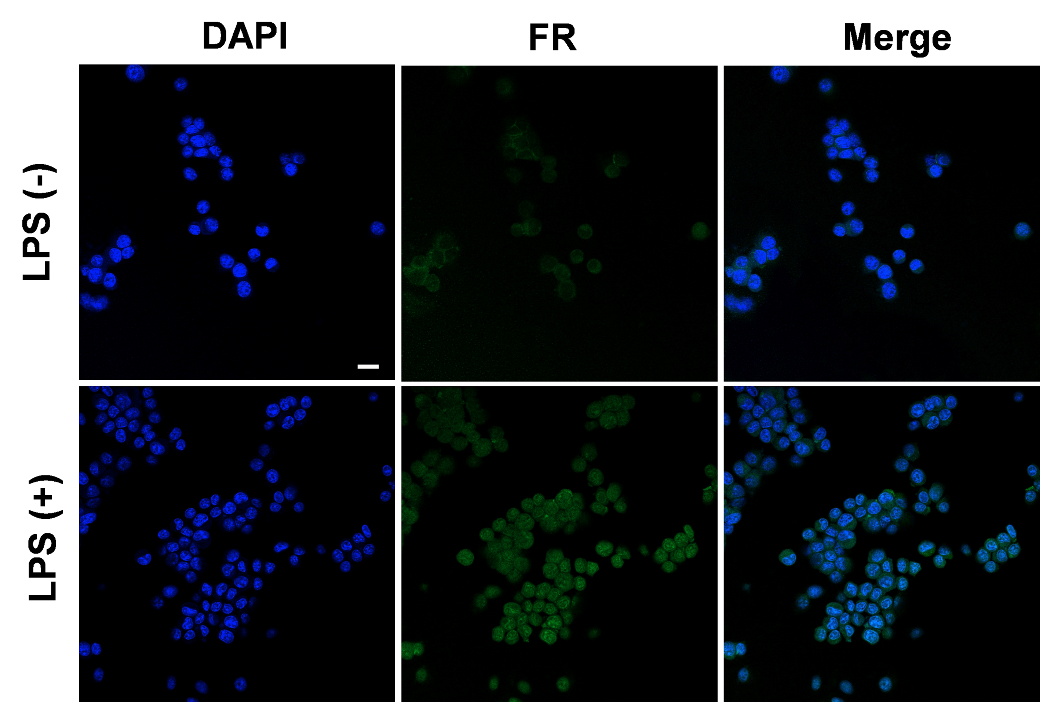


**Figure S1** Immunofluorescence staining of folate receptor (FR) expression in macrophages without and with LPS treatment as detected by LSCM. Scale bar represents 10 μm.


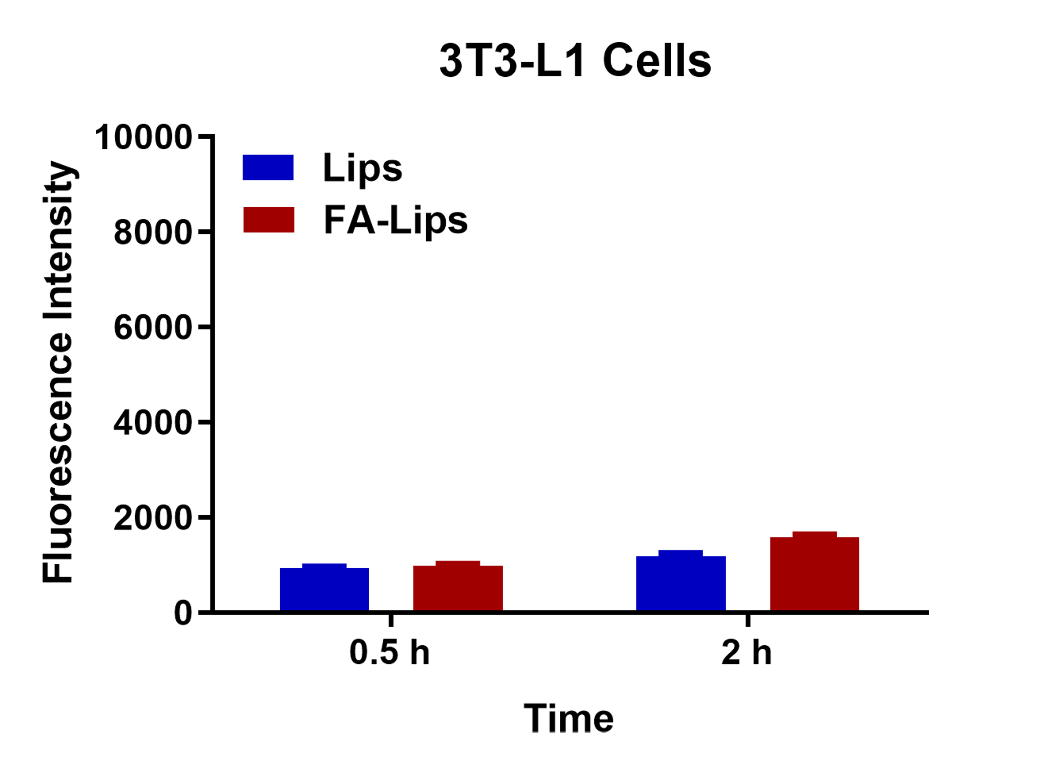


**Figure S2** Cellular uptake of Lips/DiD and FA-Lips/DiD in 3T3-L1 cells.


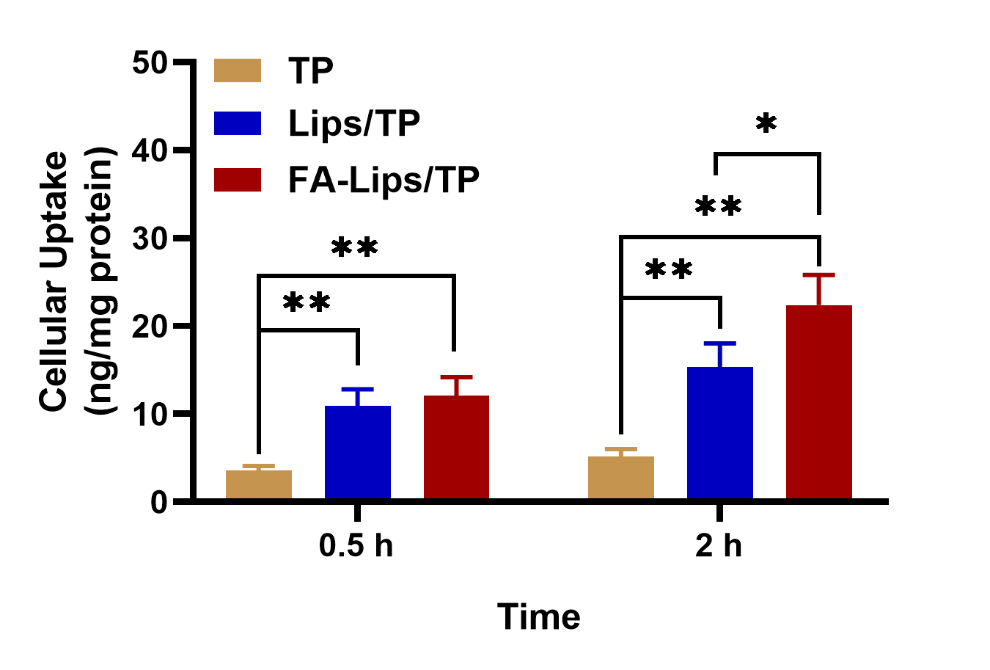


**Figure S3** Cellular uptake of TP solution, Lips/TP and FA-Lips/TP in LPS-stimulated RAW 264.7 cells. * *p* < 0.05; ** *p* < 0.01.


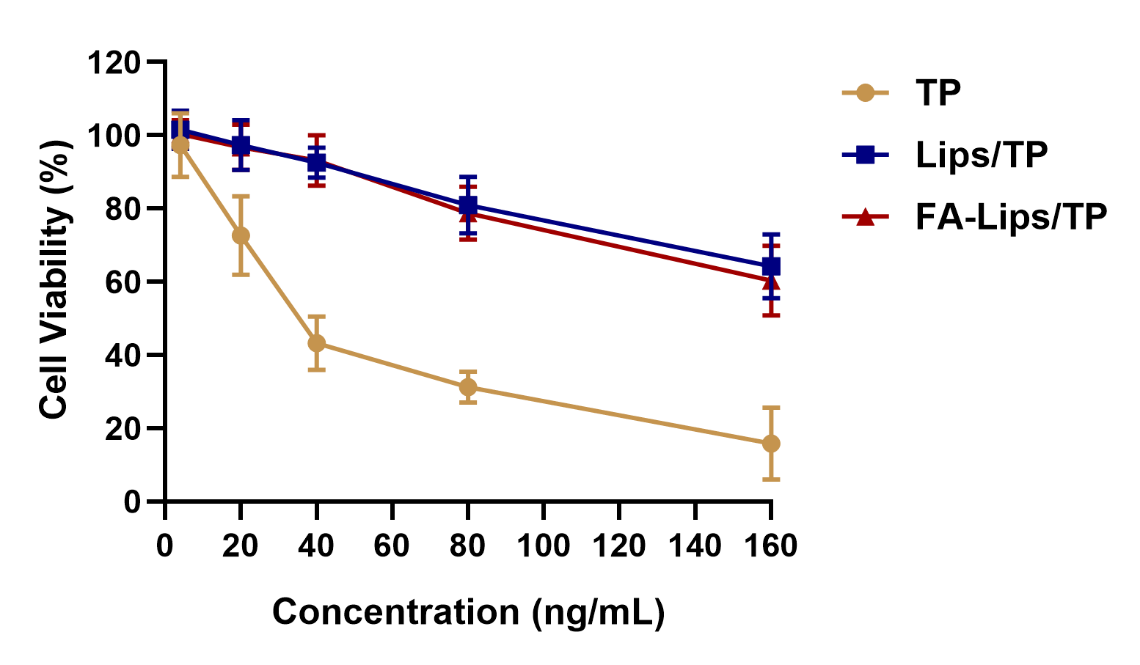


**Figure S4** Cell viability of RAW 264.7 cells after 24 h of treatment of TP solution, Lips/TP and FA-Lips/TP with varying concentrations.


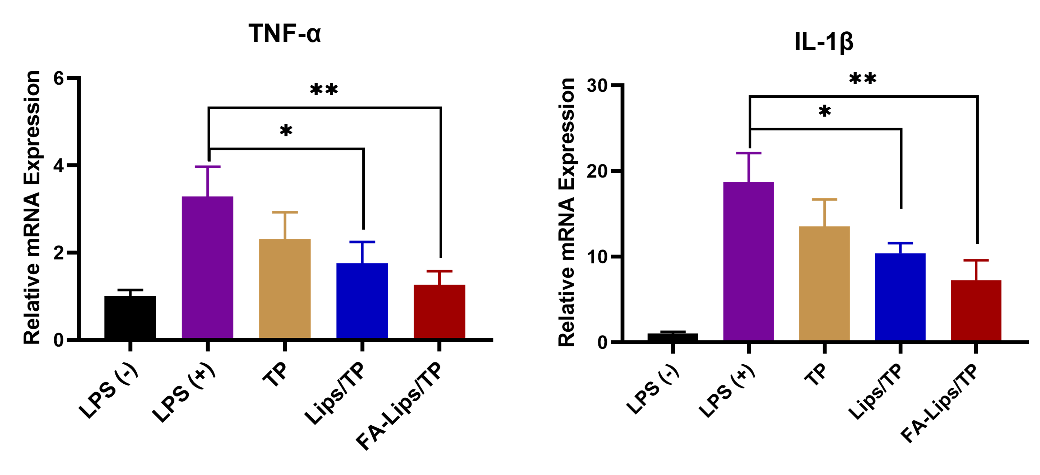


**Figure S5** Expression of TNF-α and IL-1β levels in LPS-stimulated RAW 264.7 cells after treating with TP solution, Lips/TP and FA-Lips/TP. * *p* < 0.05; ** *p* < 0.01.


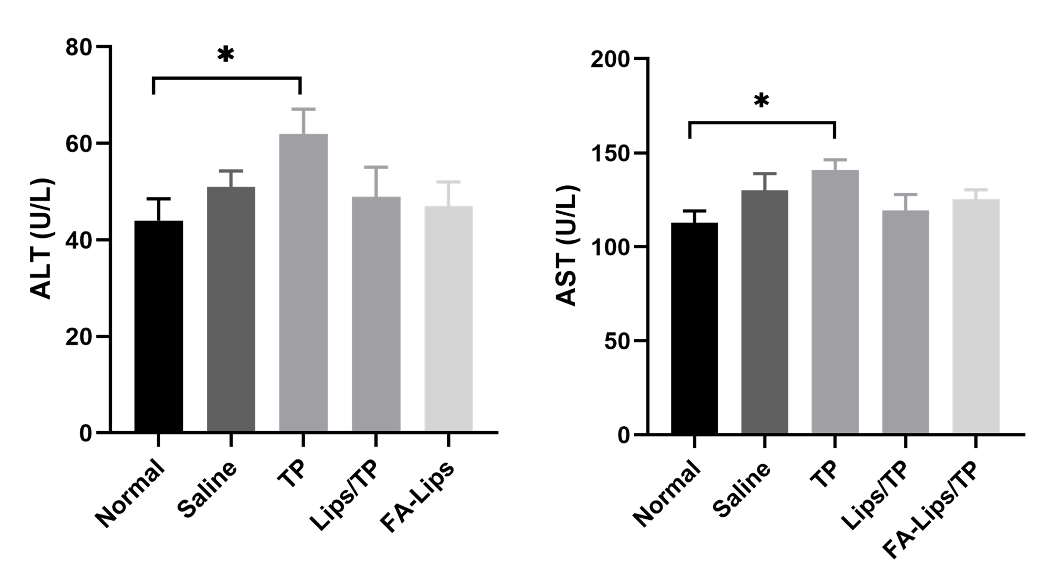


**Figure S6** serum ALT and AST levels in AIA rat model after the treatment. * p < 0.05.


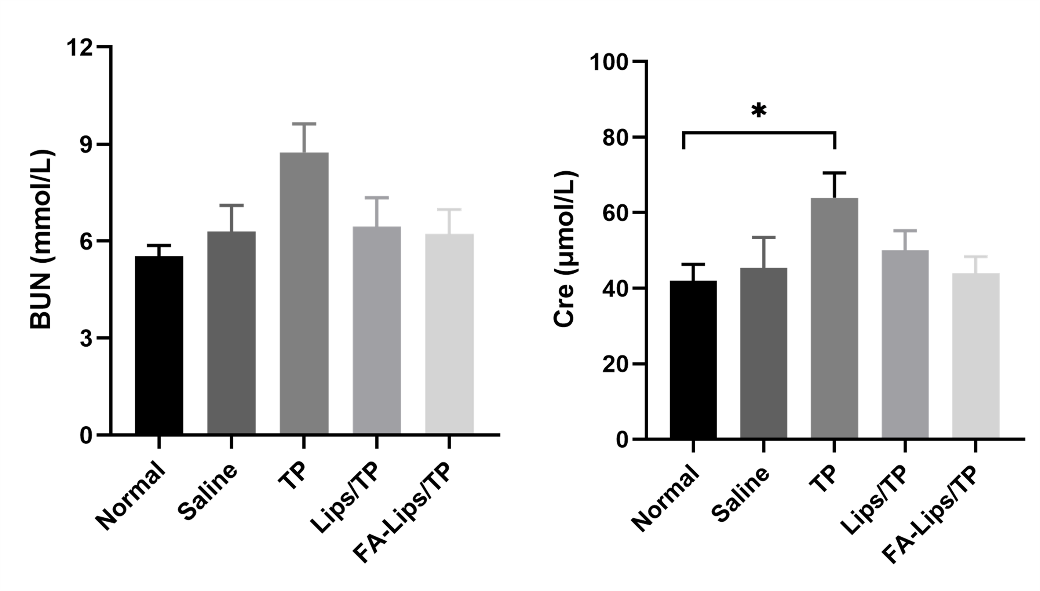


**Figure S7** serum BUN and Cre levels in AIA rat model after the treatment. * p < 0.05.
